# Rapid Profiling of the Volatilome of Cooked Meat by PTR-ToF-MS: Underlying Latent Explanatory Factors

**DOI:** 10.3390/foods9121738

**Published:** 2020-11-25

**Authors:** Giovanni Bittante, Qianlin Ni, Iuliia Khomenko, Luigi Gallo, Franco Biasioli

**Affiliations:** 1 Department of Agronomy, Food, Natural Resources, Animals, and Environment (DAFNAE), University of Padova, Viale dell’Università 16, 35020 Legnaro, Italy; giovanni.bittante@unipd.it (G.B.); qianlin.ni@studenti.unipd.it (Q.N.); 2 Department of Food Quality and Nutrition, Research and Innovation Centre, Fondazione Edmund Mach (FEM), Via E. Mach 1, 38010 San Michele all’Adige, Italy; iuliia.khomenko@fmach.it (I.K.); franco.biasioli@fmach.it (F.B.)

**Keywords:** cooked meat aroma, meat odor fingerprint, volatile organic compounds

## Abstract

Volatile organic compounds (VOCs) are important contributors to meat aroma and are variably correlated with each other. To study the sources of variation and the correlations among meat VOCs, meat cuts from five animal species/categories (chicken, turkey, pork, veal, and beef; two animals/species/retailer: 100 meat cuts) were obtained by 10 retailers. Each cut was processed into four burgers, two of which were grilled and two were cooked in a water bath (400 meat burgers). VOCs were detected by Proton-Transfer-Reaction Time-of-Flight Mass-Spectrometry (PTR-ToF-MS). From these, 129 peaks were selected, of which 72 were tentatively identified as relevant VOCs. Pearson correlations revealed a large number of positive and negative relationships among the VOCs. A multivariate statistical analysis revealed that 87% of the matrix covariance was explained by 17 independent Latent Explanatory Factors (LEFs), which have been described and characterized. LEFs identified may be valuable tools for reducing the dimensionality of results from VOC analyses and can be useful for better understanding and interpreting the variation in the meat aroma profile, although further study is required to characterize their sensory meaning.

## 1. Introduction

The intense flavor of cooked meat, which is highly appreciated by consumers, is generated during the cooking process through a complex series of chemical reactions between precursors, intermediate reaction products, and degradation [1,2,3]. Volatile organic compounds (VOCs) are a set of numerous low molecular-weight high-vapor pressure compounds that belong to different compounds which play a major role in the formation of aroma of food [4]. Particularly during and after the cooking process, many compounds in the meat are modified and dispersed in the air to form various odors, which are important criteria for evaluating meat quality [5] and may influence customer acceptability [6]. A foodomic approach requires the simultaneous, typically untargeted, analysis of the complex VOCs mixture of a food sample and modern analytical techniques might rapidly (high throughput) provide wide fingerprinting with an increasing number of features.

Several techniques are used for evaluating the VOC profiles of meat and meat products. Gas-chromatographic (GC) methods are the reference tools for VOCs identification and quantification and, in particular, for head space analysis [7]. However, the intrinsic long analysis time of GC based methods is often a limitation for practical application and high-throughput metabolomics. For this reason, faster and less invasive methods such as electric nose (e-nose) [8] and direct injection mass spectrometric methods such as PTR-MS are gaining popularity despite their limited identification capability [9,10,11]. The e-nose operates on a principle similar to the human nose using a set of chemical sensors [12] that are capable of recognizing simple or complex odors [13], but it cannot identify chemical compounds [14]. PTR-ToF-MS (proton-transfer-reaction time-of-flight mass-spectrometry) is characterized by high sensitivity, rapid analysis, and the possibility of simultaneous monitoring of a wide set of VOCs [11,15].

The number of VOCs analyzed in every meat sample could be in the hundreds [3]. Moreover, the absolute or relative concentrations of VOCs in meat are often not independent of each other, but are variously correlated, positively or negatively, so that the variations in groups of VOCs, some of them large, are interdependent and respond to common drivers [5,6]. These correlations reflect common metabolic pathways of meat, animal species differentiation, processing, packaging, and cooking procedures [2]. This means that in order to understand, interpret, and represent the variation in the meat aroma profile as a whole, we need to reduce the number of variables involved and identify the independent latent drivers of the many interrelated VOCs, goals that can be approached with multivariate statistical analyses. However, research so far has often been limited to studies of one animal species, animals with a common (experimental) origin, the same slaughtering procedure and processing and storage of cuts, and a single cooking method, etc. [16], with the consequence that they help us understand a very specific situation, but lack generalizability to a context that would be useful to the meat industry.

The aims of this study, therefore, were: (a) to obtain a balanced collection of meat samples from different animal species, origins, slaughter procedures, processing methods, retail environments, and cooking methods; (b) to analyze the cooked meat samples using PTR-ToF-MS; (c) to study the correlations among the VOCs both quantitatively and qualitatively; (d) to identify and interpret the latent explanatory factors (LEF) underlying the phenotypical measurements of individual VOCs. The novelty of this study relies on the holistic approach of considering contemporarily all the major sources of variation of meat aroma and their interactions and the attempt of extracting a limited number of comprehensive interpretive tools that could be used in different conditions, meat types, and cooking methods.

## 2. Materials and Methods

### 2.1. Experimental Design

This research is part of a wider project aimed at comparing the VOC profiles of meats from different species, of which the results are reported in a parallel study [17].

An experimental design for this study was planned with the aim of collecting meat samples that are representative of the major species and categories used for meat production according to a wide variation of retailers, animals within species/category, and cooking methods. With this purpose, meat samples originated from chicken, turkey, pork, veal, and beef were collected from 10 retailers. For each retailer, meat samples that originated from 2 animals per species or category were collected, so that a total of 100 animals were sampled (10 retailers × 5 species or category × 2 animals within species or category). We prepared 4 burgers for each animal, for a total of 400 burgers, which were cooked according to 2 different cooking methods (2 burgers/animal/cooking method).

### 2.2. Sampling, Processing, Cooking, and Analysis of Meat Samples

Ten cuts of about 600–800 g each (two cuts from two animals per species/category) were obtained every week (session) from a different retailer (10 retailers/sessions). The samples were obtained from the breast cut (*Pectoralis major* and *minor)* of poultry species and the loin cut (*Longissimus lumborum)* of mammals. Packaged meat cuts were obtained from the self-service refrigerated meat displays of supermarkets (5 sessions), whereas the meat from the local butchers (5 sessions) was freshly cut and packaged according to local practice. All retailers were regularly monitored by the local public health agency and each cut was aged according to local customs (about two weeks for beef and a few days for the other species/categories at 4–8 °C). The samples were kept in transportable refrigerators at 4 °C and shortly after collection were transferred to refrigerators in the meat laboratory of the University of Padova’s DAFNAE department (Padova, Italy).

Physical analyses of the fresh meat, sampling of meat aliquots for chemical analyses, grinding of meat samples, preparation and cooking of burgers, physical analyses of the cooked burgers, sampling of aliquots from the cooked burgers for VOCs, and other chemical analyses were carried out on all samples in a given session the day after the samples were obtained from the retailer. Briefly, each meat cut was trimmed and ground, then made into 4 burgers (meat patties) using a burger press (1.1 cm thickness), which were weighed (110 ± 2 g) and then cooked. Two burgers from each meat sample (2 burgers × 2 animals × 5 species/categories = 20 burgers per session) were sealed in polyethylene bags and cooked in a water bath preheated at 75 °C to an internal temperature of 70 °C [18]. The other two patties from each meat sample were dry-heat cooked on electric griddle at 163 °C for 3 min per side, until a target internal temperature of 70 °C [19].

After weighing the fresh meat samples, the pH of the raw meat was measured using a Sension+ pH-meter (HACH, Milano, Italy) equipped with a glass 5053T electrocode, Lab color traits were measured on both the raw meat and the cooked burgers (Minolta CM-600d spectrophotometer; Konica Minolta Sensing Americas, Inc; Ramsey, NJ, USA), while shear-force was measured on cooked patties using a multi-bladed Allo-Kramer shearing device [19] equipped with 10 blades attached to a Lloyd (Bognor Regis, UK) LS5 AMETEK testing machine. Minced raw meat samples were also analyzed for chemical composition according to Association of Official Agricultural Chemists (AOAC) [20]. After cooking, 3 g of meat were taken from the center of each burger and placed into glass vials (20 mL, Supelco, Bellefonte, PA, USA), capped with PTFE/Silicone septa (Supelco) and stored at −80 °C until VOC analysis.

### 2.3. Analysis of the Volatile Organic Compounds

VOCs were analyzed with a proton-transfer-reaction time-of-flight mass-spectrometer (PTR-ToF-MS, 8000 Ionicon Analytik GmbH, Innsbruck, Austria) coupled with a multipurpose GC automatic sampler (Autosampler, Gerstel GmbH, Mulheim am Ruhr, Germany) at the Food Quality and Nutrition Department of the Edmund Mach Foundation (San Michele all’Adige, Trento, Italy). The procedure that followed is described in a previous study on other food samples [21]. Briefly, the vials were thawed at room temperature for 6 h. For each batch, a maximum of 124 samples were chosen randomly from the 400 cooked meat samples and measured in one day. The vials were kept at 4 °C prior to analysis and each vial was incubated for 20 min at 25 °C immediately before analysis. The instrumental conditions in the drift tube were as follows: drift voltage 628 V, drift temperature 110 °C, drift pressure 2.80 mbar, affording an E/N value of about 130 Townsend (1 Td = 10–17 cm^2^/V·s), where E corresponds to the electric field strength and N to the gas number density. The sampling time per channel of ToF acquisition was 0.1 ns, amounting to 350,000 channels for a mass spectrum ranging up to *m/z* = 350. Every single spectrum is the sum of 28,600 acquisitions with a duration of 35 μs each and a resulting time resolution of 1 s. Sample measurement was performed in 70 cycles resulting in an analysis time of 60 s/sample. A 4 min interval was kept between two subsequent analyses to avoid memory effects. Spectra analysis followed as in Cappellin et al. [22] and peak were extracted from the aligned spectra and their amplitude was converted in ppbv (part per billion by volume) according to [22,23].

A total of 383 mass peaks were extracted from the raw data. Of these, 129 mass peaks were selected for further analysis after the routine mass peak selection procedure, with the elimination of mass peaks related to isotopologues, of internal ions produced by PTR-ToF-MS, namely the primary ion (H3O^+^), protonated water clusters, NO^+^, O_2_^+^, and their isotopologues, and of mass peaks of which their concentration was not significantly different from blank samples. The sum of the areas of all 129 peaks was calculated and constitutes the “quantitative” data from each burger analyzed, while the area of each of the 129 peaks expressed as a fraction of their sum constitutes the “qualitative” data (or the profile). Spectrometric peaks have been tentatively identified on the basis of their sum formula, isotopic pattern, and relevant literature, if available.

### 2.4. Data Editing and Multivariate Analyses of the Latent Explanatory Factors of VOCs

Meat burger samples with abnormal VOC profiles were identified on the basis of Mahalanobis distance. Samples with Mahalanobis distances outside the interval mean ±3.0 SD were considered outliers and all their VOC contents were discarded (*n* = 11 out of 400 samples for VOC concentrations and *n* = 14 out of 400 samples for relative proportions). A preliminary analysis of the meat VOC data was carried out using a univariate hierarchical mixed model including the fixed effects of retailer type, animal species/category, and cooking method and the random effects of retailer/session within retailer type, animal within species/category, and residual. The model is described and the results summarized in the parallel study [17].

The 129 peaks of the VOC profiles of the meat burgers were correlated between each other both as (quantitative) concentrations and as (qualitative) proportions. Latent explanatory factor (LEF) analysis was used to summarize the interrelated measured variables in a small number of unmeasured latent independent variables (factors). The analysis consisted of three steps: (1) extraction of factors with the minimum number of uncorrelated latent factors explaining the greatest proportion of common variance, (2) factor rotation until each factor was defined by relatively few variables with high loadings, and (3) biologic interpretation of the factors based on the strength of the loadings of the variables.

Factor analysis was conducted using SAS PROC FACTOR (SAS 9.4, SAS, Cary, NC, USA) with the Varimax rotation. The eigenvalues of the factors and the communality values for the measured variables after rotation were also obtained. The scores of each burger sample for each of the 17 LEFs obtained were then analyzed using the model previously described for individual VOCs [17].

## 3. Results

The descriptive statistics of the raw and cooked meat samples are summarized in Table 1.

The PTR-ToF-MS yielded a total of 383 peaks from the cooked burger samples. Peaks with a spectrometric area greater than 1 ppbv were selected, while the isotopologues and the peaks of possibly interfering ions were excluded, leaving 129 peaks for the statistical analysis. The descriptive statistics of these PTR-ToF-MS spectrometric peaks are summarized in Table 2.

The most intense peak was *m/z* 55.050 with a mean value of 182.54 µg/L, representing about 20% of the sum of all the VOCs, followed by *m/z* 43.018, 41.038, and 49.008 with mean values of 77.19, 76.80, and 68.85 µg/L, respectively, and then by *m/z* 101.097 with a mean value of 48.30 µg/L. We tentatively identified these spectrometric fragments as butanal (fragment at *m/z* 55.050), alkyl fragments (*m/z* 43.018 and *m/z* 41.038), methanethiol (*m/z* 49.008), and hexanal and/or hexan-1-one and/or hexan-2-one (*m/z* 101.097). The sum of these five VOCs represented half of the total VOCs released from the cooked meat. Another 17 peaks were of medium intensity, with mean values ranging from 32.12 µg/L to 11.53 µg/L, whereas the other 106 peaks related to compounds present in low amounts (<10 µg/L), with mean values ranging from 8.9 µg/L to 0.006 µg/L.

**Figure 1:**
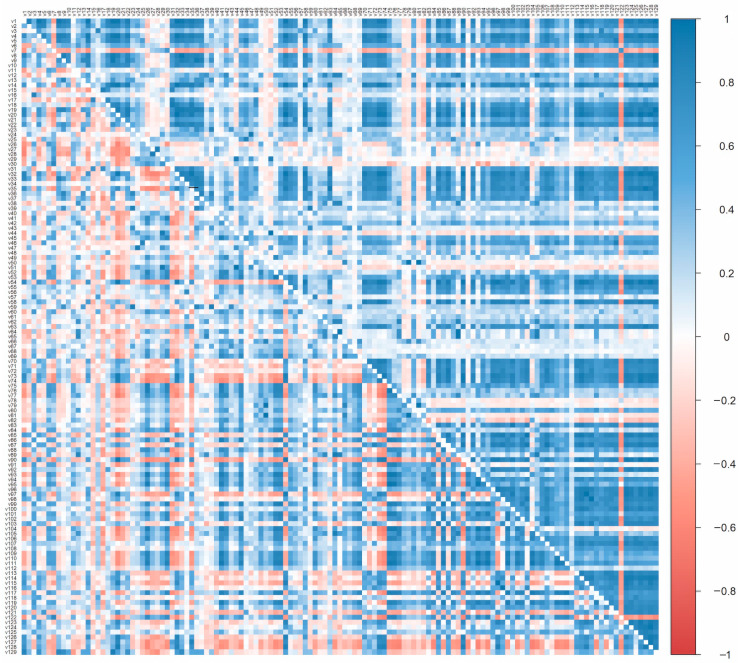
Heat map of Pearson correlations between the individual VOCs of cooked meats expressed quantitatively as concentration in the head-space (above the diagonal) and expressed qualitatively as proportion of their concentration sum (below the diagonal).

There were many positive and negative correlations among the proportions of each VOC of their sum. As can be seen from the heat map (Figure 1), the correlations were mainly positive for the quantitative VOC data because for the majority of individual VOCs, the meat samples that released the greater sums of concentrations of all VOCs tended to outnumber those releasing the lower sums of concentrations. On the other hand, the qualitative data (proportions of each VOC of their sum) tended to exhibit smaller correlations that were more equally divided between positive and negative.

To avoid overfitting of the correlations due to the effect of the overall quantity of VOCs, the multivariate latent explanatory factor analysis was carried out separately on each dataset, although only the results from the qualitative profiles are shown in Table 3 and discussed here.

The latent explanatory factor analysis allowed us to condense 87% of the entire matrix covariance into 17 latent independent factors. As can be seen from Table 3, the first latent explanatory factor (LEF-1) summarizes more than one third of the total covariance and is characterized by 48 of the 129 individual VOCs (47 with a positive loading >+0.5 and 1 with a negative loading <−0.5). The second (LEF-2) was based on 28 VOCs and explained about one fifth of the total covariance, while LEF-3 was based on 13 VOCs and explained about one tenth of the covariance. Another 8 LEFs (LEF-4 to LEF-11) were based on seven to four VOCs each and explained 7.6% to 3.1% of the total covariance (Table 3). The loadings and communalities of the 11 major LEFs are summarized in Table 4. Another six minor LEFs (LEF-12 to LEF-17) each explained 1.6% to 2.9% of all covariance (Table 3): in four cases, these were “one-VOC/one-LEF”, while only LEF-13 was based on two VOCs and LEF-17 on none. The loadings of these minor LEFs are given in Supplementary Table S1.

## 4. Discussion

### 4.1. Characterization of the Aromatic Profile of Meat

The number of mass peaks measured by PTR-ToF-MS (129 selected here out of a total of 383 extracted from the raw data) was much larger than the number of the compounds usually measured by SPME-MS. In our study, the dominant VOCs on all the various types of meat were tentatively identified (t.i.) as butanal (*m/z* 55.050), methanethiol (*m/z* 49.008), some common fragments (*m/z* 41.038, *m/z* 43.018), pentanal or pentanone (*m/z* 69.070), acetoin (3-hydroxy-2-butanone), ethyl acetate or butanoic acid (*m/z* 89.060), and unknown peaks (*m/z* 101.097). Of these, butanal (*m/z* 55.050) was the most abundant VOC in the meat samples from all the species. It is formed mainly by β-oxidation of unsaturated fatty acids [21] and contributes a malty, green, roast odor [6]. Methanethiol (*m/z* 49.008) was the second most abundant VOC. Belonging to the sulfurous compounds, it is formed by degradation of the amino acids and depending on the concentration and on the interaction with other matrix constituents, it can provide a vegetable, sulphurous, boiled cabbage, or eggy note [21]. The third most abundant VOC differed in the different species: on average, it was dimethyl sulfide or ethanthiol (*m/z* 63.026). Dimethyl sulfide is formed by catabolism of the amino acids and contributes a rotten garlic odor [21]. Hexanal, hexan-1-one, or hexan-2-one (*m/z* 101.097) was also an important VOC. Shi and Ho [24] identified hexanal as the most abundant aldehyde in chicken, but it does not seem to be present in the other species. Hexanal is formed particularly by primary oxidation products of linoleic acid and contributes a green, fruity odor [25]. Other abundant VOCs were *m/z* 101.097 and *m/z* 61.035: the latter was identified as a group of VOCs that includes acetic acid, a fragment of butyl acetate, 2-methylbutyl acetate, or isobutyl acetate and of these, Lawrie [26] has confirmed butyl acetate and isobutyl acetate as being present among meat VOCs.

The very high number of individual VOCs found in the headspace of the vial containing the meat samples and the numerous and complex relationships among them make summarizing the VOCs and describing the aroma of cooked meat a very difficult task [16]. To do so, it is necessary to reduce the dimensionality of the database and extract from the VOC profile a few independent latent explanatory factors explaining the major part of the variation in the VOCs.

### 4.2. Latent Explanatory Factors (LEF) of Cooked Meat Odor

Multivariate analyses of the entire VOC dataset helped us reduce the dimensionality of odor descriptors from 129 individual interrelated VOCs to just 17 latent explanatory factors (LEFs) independent of each other. All together, they absorbed 87.14% of the entire covariance matrix among all the individual VOCs. Moreover, these 17 LEFs explained the major part of the variance in all VOCs. The communality was, in fact, >0.70 for all VOCs, except for four where it was >0.55 (Table 4). Only 6 out of the 129 VOCs (Table 4 and Supplementary Table S1) did not characterize any LEF with no loading >+0.5 or <−0.5; these had one or two loadings >+0.45 and/or <−0.39 and their communality ranged from 0.64 to 0.76.

The LEFs can be a very useful statistical tool for identifying the groups of volatile peaks with common drivers and then to condensate the major part of the variability of the highly correlated 129 peaks studied in few independent latent factors. On the other hand, they cannot give, at the moment, a precise idea of the specific meat odor that they represent. The tentative characterization of their effect on meat aroma, in absence of specific research, could only be based on the known odorous characteristics of some of their most representative VOCs. However, it should be kept in mind that combining different volatile substances, changing their proportions, and interacting with different base matrices leads to changes in the sensorial stimulation of the human nose and mouth. Only specific research that tries to correlate the variation in LEFs intensity with variation in sensory descriptors of meat will be able to characterize the odorous properties of each LEF in itself (and not of their individual peaks).

#### 4.2.1. LEF-1 “Meaty, Fresh, Fruity, Pungent, Garlic Odors”

LEF-1 was the most important factor, explaining 35.09% of the total covariance matrix. Moreover, 48 out of the 129 individual VOCs found in meat odor had loadings >+0.5 (47 VOCs) or <−0.5 (1 VOC). The VOC that, unlike all the others, had a negative loading was *m/z* 67.021, which was present in meat in very low concentrations (Table 2). It is worth noting that only 2 VOCs characterizing LEF-1 were present in average concentrations of >10 µg/L and could therefore be considered as having high quantitative relevance for meat odor, these being *m/z* 33.034 (t.i. as methanol) and *m/z* 61.035 (t.i. acetic acid and fragment of butyl acetate, 2-methylbutyl acetate, isobutyl acetate). Both these peaks, as well as m/z 95.019 (t.i. dimethyl sulfone), *m/z* 103.048, *m/z* 115.079, *m/z* 117.092 (t.i. hexanoic acid, ethyl butanoate, methyl isovalerate), and *m/z* 121.066 (t.i. acetophenone, 4-methyl-benzaldehyde), have been detected in chicken by Franke and Beauchamp [27], Keupp et al. [28], Du et al. [29], Silvis et al. [30], Rajamäki et al. [31], and Lytou et al. [32]. Peak *m/z* 61.035 and also peaks *m/z* 75.044, *m/z* 117.092, *m/z* 125.024 (hydroxy-benzoquinone), and *m/z* 131.109 have been detected on beef [33,34]. Peaks *m/z* 34.996 (hydrogen sulfide), *m/z* 125.024, and m/z 131.109 (heptanoic acid, ethyl-2-methylbutanoate, ethyl-3-methylbutanoate, methylbutyl acetate) have been detected on pork and salami [35,36,37].

Representing the most numerous and quantitatively important group of volatile mass peaks, the LEF-1 will probably represent in some way the basic “meaty” odors that are common in most of the cooked meats. Some additional information could be obtained considering the known odor of the most relevant mass peaks represented in LEF-1. Some of these peaks contribute to a fruity-like odor (*m/z* 61.035, *m/z* 75.044, *m/z* 103.048, *m/z* 115.079, *m/z* 117.092, *m/z* 121.066) and others (*m/z* 33.034, *m/z* 34.996, *m/z* 95.019) a pungent, unpleasant, garlic-like, off odor [2,6]. For these reasons, we have tentatively described the effect of this LEF on meat aroma as “fresh, fruity, pungent, garlic odor”, even if we are fully conscious of the weakness of this definition and the need for specific research connecting the LEF to odor descriptors obtained by sensory analysis on the same meat samples analyzed for VOCs.

#### 4.2.2. LEF-2 “Green, Leafy, Nutty, Waxy, Fruity Odors”

LEF-2 is very important, explaining 20.77% of total co-variance and characterized by 28 peaks, of which those that could be considered relevant are *m/z* 53.039 and *m/z* 69.070 (t.i. pentanal, pentenol). Several peaks characterizing this LEF were found in meat in previous studies. Mayr et al. [38] identified acetaldehyde in beef (*m/z* 46.034), while peak *m/z* 67.055 was identified on poultry meat (chicken and turkey) by Brunton, Cronin, Monahan, & Durcan [39]. Du et al. [29] identified pentanal, which contributes a fermented, bready, fruity, nutty, berry odor and pentanal was also detected on lamb meat by Gravador et al. [40]. Other peaks characterizing LEF-2—*m/z* 99.082 (2-hexenal, trans-2-hexenal, 2-hexanone or hexanone acid), *m/z* 101.097 (hexanal, hexan-1-one, hexan-2-one), *m/z* 115.113 (heptanal, heptan-2-one), *m/z* 143.146 (methyloctanol, nonanal, nonan-2-one), and *m/z* 165.161 (2-dodecenal)—have been detected on beef, pork, and poultry meats [41,42,43,44]. Peak *m/z* 101.097 has been identified as hexanal [6], or hexan-2-one [45], but they have similar leaf-like, green odors [30]. Peak *m/z* 115.113 has been identified as heptan-2-one [46] with a fruity, fatty, sweet odor [30]; *m/z* 143.146 has been identified as nonanal [6] or nonan-2-one [47] with a waxy, plastic odor [48]; and *m/z* 165.161 has been identified as 2-dodecenal [49]. Given the presence of a lot of peaks with different odorant properties and in different proportions, we cannot associate this LEF with a particular odor. More specific studies are needed in the future.

#### 4.2.3. LEF-3 “Fusel-Like Pungent Odor”

LEF-3 explained 9.5% of total variance and was characterized by 13 VOCs, many of which could be considered relevant for meat aroma: *m/z* 41.038 (common fragment), *m/z* 43.055 (common fragment), *m/z* 53.003, *m/z* 57.034 (t.i. propenal), *m/z* 57.070 (t.i. butanol, isobutanol), and *m/z* 71.085 (t.i. methyl butanol, pentanol). It is worth noting that butanol has a harsh fusel/pungent odor [50] and also pentanol has a characteristic fusel-like/alcoholic odor [51].

#### 4.2.4. LEF-4 “Malty, Butter, Roast Odors”

LEF-4 explained 7.6% of total variance and was based on seven significant VOCs, of which the relevant ones were *m/z* 43,018 (common fragment) and *m/z* 89.060 (t.i. acetoin (3-hydroxy-2-butanone), ethyl acetate, butanoic acid). Peak *m/z* 71.049 has also been detected on beef [6] and salami [36], contributing a malty, green, roast aroma. Peak *m/z* 87.044 (2,3-butanedione, diacety) has been detected on chicken [28] and cheese [21] and has been identified as contributing a butter-sweet aroma. Peak *m/z* 89.060 (butanoate acid, acetoin (3-hydroxy-2-butanone), ethyl acetate) has been detected on chicken and beef [28,52], the former contributing a butter, caramel aroma [21] and the latter, a fruity aroma [30,53].

#### 4.2.5. LEF-5 “Aromatic Odor”

This LEF explained 4.7% of total variance and was based on five significant VOCs: *m/z* 78.979, *m/z* 79.055 (benzene, aromatic fragment), *m/z* 91.059 (diethyl sulfide), *m/z* 106.079, *m/z* 107.086 (xylene), although none could be considered relevant for meat aroma. Benzene has a gasoline-like and pleasant aromatic odor [54]; diethyl sulfide has a garlic-like odor [55].

#### 4.2.6. LEF-6 “Fatty, Grassy, Plastic Odors”

This LEF explained 4.4% of total variance and was based on seven significant VOCs, but here, too, none could be considered relevant for meat aroma. Lustig and Schuetz [56] detected *m/z* 28.032 on meat and thought that it just came from the meat packaging, but in our study, we detected m/z 28.032 on all species without packaging. Peaks *m/z* 111.118 (octenol, octanal) and *m/z* 115.113 (heptanal, heptan-2-one) have been detected on beef [46,48], with a similar fruity, fatty, sweet odor [30]. Peak *m/z* 143.146 (methyloctanol, nonanal, nonan-2-one) has also been detected on beef [6,47]. Nonanal also contributes a fatty, grassy odor, whereas nonan-2-one gives a plastic, earthy odor. Peak *m/z* 129.128 (octanal, octanone) has been detected on pepper and is characterized by a mushroom-like odor [30]. Peak *m/z* 143.146 (methyloctanol, nonanal, nonan-2-one) has also been detected on beef [6,47], imparting a fatty, grassy, plastic, earthy aroma.

#### 4.2.7. LEF-7 “Unknown Odor”

This LEF explained 4.4% of total variance and was based on seven significant VOCs, of which one (*m/z* 75.944) is quantitatively relevant, even though it is of unknown odor. Peak *m/z* 59.967 has been identified in chicken as giving a fruity odor [28]. We also found some peaks not previously identified in meat (*m/z* 63.947, *m/z* 77.976, and *m/z* 79.938), inter-correlated, and mainly of unknown origin and odor.

#### 4.2.8. LEF-8 “Fruity Odor”

This LEF explained 3.8% of total variance and was based on four significant VOCs, none of which could be considered relevant for meat aroma in quantitative terms. Peaks m/z 69.034 (furan) and *m/z* 84.044 (hexanal, hexenol) have been detected in beef and poultry [29,33,53,57], as has peak *m/z* 87.080 (pentanal, pentanone) [48,58], all of them giving a fruity odor, except 1-penten-3-ol, which gives a gasoline odor. Aside from confirming previous results, we also identified two other peaks in LEF-8 that had not previously been associated with meat (*m/z* 86.970 and *m/z* 87.080, pentanal, pentanone).

#### 4.2.9. LEF-9 “Fragrant-Aromatic Odor”

This LEF explained 3.5% of total variance and was also based on four significant VOCs: *m/z* 29.039 (common fragment), *m/z* 47.049 (ethanol), *m/z* 85.014 (thiophene), and *m/z* 147.130, none of which could be considered relevant for meat aroma. Ethanol has a fragrant odor [59] and thiophene an aromatic odor [60].

#### 4.2.10. LEF-10 “Pungent, Irritating Odor”

This LEF explained 3.4% of total variance and was again based on four significant VOCs, although only two of them could be considered relevant for meat aroma: *m/z* 31.019 (t.i. formaldehyde) and *m/z* 60.053 (acetone isotopologue). Formaldehyde has been detected on cheese, but not on meat [61], and imparts a pungent odor. Propan-2-one is characterized by a pungent, irritating, floral, cucumber-like odor in cheese [21]. The other two peaks, which were found in much lower concentrations, were *m/z* 77.059 (propylene glycol) and *m/z* 159.137 (nonanoic acid, 3-methylbutyl butanoate).

#### 4.2.11. LEF-11 “Acrid Sulfurous, Roast Odor”

LEF-11 explained 3.1% of total variance and was also based on four significant VOCs, but this LEF is very important as three of the four VOCs could be considered relevant for meat aroma: *m/z* 46.996 (t.i. thioformaldehyde), *m/z* 49.008 (methanethiol), and *m/z* 55.050 (t.i. butanal). Methanethione and methanethiol have been identified in the meat of different species [62] and contribute important sulfurous notes. Butanal has been identified in beef [6] as giving an acrid, malty, green, roast odor. We also found a less important peak (*m/z* 67.021) associated with the other three.

#### 4.2.12. Minor LEFs (LEF-12 to LEF-17)

Six other minor LEFs (LEF-12 to LEF-17) explained from 2.9% to 1.6% of total variance. One of these (LEF-13) was based on 2 VOCs, four of them (LEF-12, LEF-14, LEF-15, and LEF-16) on one significant VOC, and one (LEF-17) had no VOC, with a loading reaching the threshold for significance.

It is worth noting that of the two peaks characterizing LEF-13, one of them, *m/z* 63.026, was identified as dimethyl sulfide [28], which gives a sulfurous, rotten garlic odor [21] and could be considered relevant for meat aroma. The other peak, *m/z* 62.023, has been identified as nitromethane in pepper [30].

## 5. Conclusions

The VOC profile of cooked meat is very complex as it consists of hundreds of volatile substances. Using PTR-Tof-MS and after deleting all the mass peaks with very low concentrations, we still detected 129 mass peaks on chicken, turkey, pork, veal, and beef meat. The thousands of significant correlations among the concentrations of volatile compounds make it impossible to characterize meat aroma unless we extract a modest number of independent latent explanatory factors representing a large proportion of the complex meat aroma. The LEFs identified are potentially valuable tools for reducing the volume of the results from VOC analysis and may be useful for representing the variation in the meat aroma profile, although further study is required to characterize their sensory meaning.

## Figures and Tables

**Table 1:** Mean, standard deviation (SD), minimum (Min), and maximum (Max) of selected traits of meat sampled before (20 animals per species/category) and after processing and cooking (80 meat patties per species/category).

Parameters:	Mean	SD	Min	Max
Raw patty composition (g/100 g):				
- moisture	74.05	1.54	69.36	77.92
- protein	22.12	1.33	18.23	25.04
- lipid	2.12	1.64	0.33	11.06
- ash	1.13	0.08	0.96	1.58
Raw patty pH:	5.77	0.21	5.52	6.82
Raw patty color:				
- lightness, L*	46.36	5.83	25.94	59.60
- redness, a*	5.72	5.72	−0.80	22.48
- yellowness, b*	14.66	4.17	6.69	26.60
Cooked patty color:				
- lightness, L*	64.57	12.01	29.16	82.51
- redness, a*	5.05	3.63	0.47	20.45
- yellowness, b*	20.66	5.70	11.66	40.50
Cooked patty shear force, N/g	19.8	5.2	9.9	42.5

**Table 2:** Mean, standard deviation (SD), minimum (Min), and maximum (Max) of PTR-ToF-MS mass peaks expressed as percentage proportion of the sum of all VOCs and of their Pearson correlation coefficient (*r*) with the sum of all VOCs.

	*m/z*	Raw Formula	Tentative Identification	Mean	SD	Min	Max	*r*
v1	26.016	C_2_H_2_^+^	Common fragment	0.89	0.24	0.17	1.52	0.40
v2	28.032	C_2_H_4_^+^		0.08	0.03	0.03	0.17	0.15
v3	29.039	C_2_H_5_^+^	Common fragment	0.27	0.12	0.13	0.77	−0.56
v4	29.060			0.03	0.01	0.01	0.05	0.10
v5	31.019	CH_2_OH^+^	Formaldehyde	1.57	0.48	0.60	3.44	0.16
v6	33.034	CH_4_OH^+^	Methanol	3.49	1.72	1.27	26.97	−0.56
v7	34.996	H_2_SH^+^	Hydrogen sulfide	1.27	1.51	0.01	7.94	−0.52
v8	38.018			0.05	0.02	0.01	0.09	0.19
v9	41.038	C_3_H_5_^+^	Common fragment	8.42	2.76	1.80	20.81	0.10
v10	42.011			0.98	0.25	0.26	1.83	−0.16
v11	42.034	C_2_H_3_NH^+^	Acetonitrile	1.73	1.55	0.20	10.30	−0.40
v12	43.018	C_2_H_3_O^+^	Common fragment	9.09	3.90	2.83	22.14	−0.30
v13	43.055	C_3_H_7_^+^	Common fragment	2.58	1.47	0.64	8.47	−0.15
v14	46.034	C^13^CH_4_OH^+^		0.69	0.13	0.25	1.10	0.04
v15	46.996	CH_2_SH^+^	Thioformaldehyde	1.10	0.96	0.05	5.95	0.27
v16	47.049	C_2_H_6_OH^+^	Ethanol	1.05	0.87	0.17	5.41	−0.31
v17	49.008	CH_4_SH^+^	Methanethiol	5.81	7.07	0.04	32.92	0.50
v18	52.028			0.01	0.00	0.00	0.02	−0.06
v19	53.003			1.56	0.42	0.34	2.96	0.21
v20	53.039	C_4_H_5_^+^		2.22	0.97	0.16	5.02	0.57
v21	55.050	C_4_H_7_^+^	Butanal	19.30	7.99	0.07	37.87	0.21
v22	57.034	C_3_H_4_OH^+^	Propenal, or common fragment	1.83	0.46	0.44	3.49	0.19
v23	57.070	C_4_H_9_^+^	Butanol, isobutanol	1.51	0.81	0.31	4.17	−0.25
v24	59.967			0.18	0.10	0.02	0.45	−0.28
v25	60.053	C_2_^13^CH_7_^+^	Isotope of Acetone, Propan-2-one	1.54	0.82	0.49	4.88	−0.43
v26	61.035	C_2_H_4_O_2_H^+^	Acetic acid, fragment of Butyl acetate, 2-Methylbutyl acetate, Isobutyl acetate	4.16	2.08	1.09	11.23	−0.79
v27	62.023	CH_3_NO_2_H^+^		0.05	0.03	0.01	0.27	−0.39
v28	63.026	C_2_H_6_SH^+^	Dimethyl sulfide, ethanthiol	3.03	3.23	0.13	22.19	−0.23
v29	63.947			0.00	0.00	0.00	0.01	−0.49
v30	63.986			0.05	0.03	0.02	0.20	−0.80
v31	67.021			0.01	0.01	0.00	0.04	0.66
v32	67.055	C_5_H_7_^+^	Pentenal or common fragment	0.36	0.12	0.06	0.66	0.49
v33	67.992			0.00	0.00	0.00	0.00	0.28
v34	69.034	C_4_H_4_OH^+^	Furan	0.06	0.02	0.02	0.16	−0.13
v35	69.070	C_5_H_9_^+^	Isoprene or common fragment of aldehydes, alcohols and terpenes)	2.73	0.99	0.35	6.10	0.62
v36	70.004			0.00	0.00	0.00	0.01	−0.28
v37	71.015			0.03	0.01	0.01	0.06	−0.18
v38	71.049	C_4_H_6_OH^+^	2-Butenal, Methyl vinyl ketone	0.63	0.59	0.07	2.99	−0.09
v39	71.085	C_5_H_11_^+^	Methyl butanol, Pentanol	1.78	1.02	0.20	4.62	−0.17
v40	73.065	C_4_H_8_OH^+^	2-Butanone, Butanal	3.05	2.12	0.79	13.82	−0.32
v41	75.028	C_3_H_6_SH^+^	Allyl mercaptan	0.07	0.03	0.03	0.21	−0.84
v42	75.044	C_3_H_6_O_2_H^+^	Propanoic acid, Methyl acetate	0.36	0.13	0.16	0.77	−0.67
v43	75.081	C_4_H_10_OH^+^		0.04	0.03	0.01	0.19	−0.26
v44	75.944			1.41	1.54	0.02	8.40	−0.41
v45	77.016			0.02	0.01	0.01	0.06	−0.81
v46	77.059	C_3_H_8_O_2_H^+^	Propylene Glycol	0.74	0.33	0.21	2.06	−0.02
v47	77.976			0.03	0.02	0.00	0.09	−0.29
v48	78.979			0.01	0.00	0.00	0.04	−0.12
v49	79.039	C_2_H_6_O_3_H^+^	Adduct of water and C_2_H_4_O_2_H^+^	0.12	0.06	0.03	0.34	−0.61
v50	79.055	C_6_H_7_^+^	Benzene, Aromatic fragment	0.41	0.39	0.11	2.67	−0.33
v51	79.938			0.00	0.00	0.00	0.02	−0.47
v52	80.041			0.01	0.01	0.00	0.03	−0.82
v53	81.038	C_5_H_4_OH^+^		0.05	0.02	0.02	0.12	−0.80
v54	81.071	C_6_H_9_^+^	Hexenal, common fragment	0.34	0.13	0.10	0.75	0.46
v55	82.047			0.01	0.00	0.00	0.01	−0.44
v56	84.044	C_5_^13^CH_11_^+^	Hexanal, Hexenol	0.02	0.00	0.00	0.04	−0.69
v57	85.014	C_4_H_4_SH^+^	Thiophene	0.02	0.02	0.00	0.10	−0.28
v58	85.073			0.05	0.01	0.02	0.09	−0.15
v59	85.101	C_6_H_13_^+^	Hexanol	0.21	0.15	0.02	0.73	−0.14
v60	86.022			0.01	0.00	0.00	0.02	−0.67
v61	86.970			0.01	0.00	0.00	0.03	−0.41
v62	87.044	C_4_H_6_O_2_H^+^	2,3-Butanedione, diacetyl	0.42	0.25	0.10	1.83	−0.24
v63	87.080	C_5_H_10_OH^+^	Pentanal, Pentanone	0.67	0.22	0.11	1.53	0.20
v64	88.960			0.01	0.00	0.00	0.02	−0.60
v65	89.060	C_4_H_8_O_2_H^+^	Acetoin (3-Hydroxy-2-butanone), Ethyl acetate, Butanoic acid	1.40	1.57	0.07	8.27	−0.06
v66	91.059	C_4_H_10_SH^+^	Diethyl sulfide	0.12	0.07	0.04	0.56	−0.49
v67	93.069	C_7_H_9_^+^	Toluene	0.25	0.18	0.04	1.50	−0.36
v68	95.019	C_2_H_6_O_2_SH^+^	Dimethyl sulfone	0.29	0.15	0.09	0.90	−0.63
v69	95.053			0.11	0.06	0.04	0.46	−0.74
v70	95.088	C_7_H_11_^+^	Heptenal, common fragment	0.07	0.02	0.03	0.12	0.00
v71	97.064	C_6_H_8_OH^+^	2,5-Dimethylfuran, Ethylfuran	0.07	0.03	0.02	0.17	0.45
v72	97.101			0.19	0.07	0.05	0.40	0.02
v73	99.082	C_6_H_10_OH^+^	2-Hexenal, Trans-2-hexenal, 2-Hexanone, Hexanone acid	0.07	0.03	0.01	0.15	0.53
v74	101.097	C_6_H_12_OH^+^	Hexanal, Hexan-1-one, Hexan-2-one	4.67	2.72	0.10	12.32	0.64
v75	102.026			0.01	0.00	0.00	0.02	−0.87
v76	103.048			0.02	0.01	0.01	0.05	−0.84
v77	105.041			0.01	0.00	0.00	0.03	−0.77
v78	105.069	C_8_H_9_^+^	Styrene	0.03	0.03	0.01	0.19	−0.45
v79	106.079			0.02	0.02	0.00	0.10	−0.38
v80	107.056			0.08	0.03	0.03	0.18	−0.57
v81	107.086	C_8_H_11_^+^	Xylene	0.48	0.63	0.06	4.77	−0.32
v82	109.076	C_6_H_8_N_2_H^+^	2,5-Dimethylpyrazine	0.02	0.02	0.00	0.10	−0.53
v83	109.103	C_8_H_13_^+^	Octenal, common fragment	0.05	0.01	0.02	0.14	−0.59
v84	110.969			0.00	0.00	0.00	0.01	−0.77
v85	111.118	C_8_H_15_^+^	Octenol, Octanal	0.12	0.05	0.03	0.29	0.20
v86	115.079	C_6_H_10_O_2_H^+^	Caprolactone	0.02	0.01	0.01	0.07	−0.84
v87	115.113	C_7_H_14_OH^+^	Heptanal, Heptan-2-one	0.09	0.03	0.03	0.18	0.16
v88	117.092	C_6_H_12_O_2_H^+^	Hexanoic acid, Ethyl butanoate, Methyl isovalerate and other C6 esters/acids	0.02	0.01	0.01	0.06	−0.75
v89	118.056			0.00	0.00	0.00	0.01	−0.48
v90	119.105	C_6_H_14_O_2_H^+^		0.20	0.09	0.02	0.48	0.56
v91	121.066	C_8_H_8_OH^+^	Acetophenone, 4-Methyl-benzaldehyde	0.03	0.02	0.01	0.15	−0.75
v92	121.105	C_9_H_13_^+^	Trimethylbenzene	0.01	0.00	0.00	0.04	−0.50
v93	123.050	C_4_H_10_O_2_SH^+^		0.01	0.01	0.01	0.05	−0.87
v94	123.114	C_9_H_15_^+^	Nonenal	0.01	0.00	0.01	0.03	−0.68
v95	125.024	C_6_H_4_O_3_H^+^	Hydroxy-benzoquinone	0.01	0.00	0.00	0.03	−0.86
v96	125.067			0.01	0.00	0.00	0.02	−0.45
v97	125.097	C_8_H_12_OH^+^	Octadienone	0.11	0.06	0.01	0.32	0.53
v98	125.132	C_9_H_17_^+^	Nonanal, Nonenol	0.03	0.01	0.01	0.06	0.09
v99	127.081			0.01	0.00	0.00	0.02	−0.84
v100	127.113	C_8_H_14_OH^+^	Octenal, 1-Octen-3-one	0.02	0.01	0.01	0.05	−0.36
v101	128.973			0.00	0.00	0.00	0.00	−0.83
v102	129.093	C_7_H_12_O_2_H^+^	Butyl propenoate, Allyl butyrate	0.01	0.00	0.00	0.03	−0.76
v103	129.128	C_8_H_16_OH^+^	Octanal, Octanone	0.06	0.02	0.02	0.15	0.05
v104	130.041			0.00	0.00	0.00	0.02	−0.88
v105	131.076			0.00	0.00	0.00	0.01	−0.72
v106	131.109	C_7_H_14_O_2_H^+^	Heptanoic acid, Ethyl-2-methylbutanoate, Ethyl-3-methylbutanoate, Methylbutyl acetate and other C7 esters/acids	0.00	0.00	0.00	0.01	−0.71
v107	133.112			0.01	0.00	0.00	0.01	−0.45
v108	134.975			0.00	0.00	0.00	0.00	−0.47
v109	135.043			0.00	0.00	0.00	0.01	−0.86
v110	135.087	C_6_H_14_OSH^+^	3-Mercaptohexanol	0.00	0.00	0.00	0.01	−0.80
v111	137.067			0.01	0.00	0.00	0.02	−0.88
v112	137.132	C_10_H_17_^+^	Monoterpenes	0.02	0.01	0.00	0.09	−0.54
v113	139.114	C_9_H_14_OH^+^	2,6-Nonaienal, Isophorone, Pentylfuran	0.04	0.02	0.01	0.11	0.47
v114	141.130	C_9_H_16_OH^+^	Nonenal, Nonenone	0.01	0.00	0.00	0.01	0.23
v115	143.106	C_8_H_14_O_2_H^+^	Hexenyl acetate,	0.30	0.16	0.02	1.00	0.49
v116	143.146	C_9_H_18_OH^+^	Methyloctanol, Nonanal, Nonan-2-one	0.07	0.03	0.02	0.17	0.05
v117	145.060			0.00	0.00	0.00	0.01	−0.84
v118	147.130			0.00	0.00	0.00	0.01	0.06
v119	151.120			0.00	0.00	0.00	0.01	−0.83
v120	153.131			0.00	0.00	0.00	0.02	−0.47
v121	159.137	C_9_H_18_O_2_H^+^	Nonanoic acid, 3-Methylbutyl butanoate and other C9 esters/acids	0.01	0.01	0.00	0.03	0.53
v122	160.899			0.00	0.00	0.00	0.00	−0.71
v123	161.120			0.01	0.00	0.00	0.01	0.21
v124	165.161	C_12_H_21_^+^	2-Dodecenal	0.00	0.00	0.00	0.00	0.11
v125	173.148			0.00	0.00	0.00	0.00	−0.27
v126	175.122			0.00	0.00	0.00	0.00	0.26
v127	187.169	C_11_H_22_O_2_H^+^	Methyl caprate, Ethyl nonanoate and other C11 esters/acids	0.00	0.00	0.00	0.00	0.73
v128	201.182	C_11_H_24_O_2_H^+^		0.00	0.00	0.00	0.02	0.69
v129	241.959			0.00	0.00	0.00	0.01	0.24

**Table 3:** Latent explanatory factors (LEFs) of the relative percentage incidence of volatile organic compounds (VOCs) on cooked meat patties.

Items:	Eigen Value	Variance Explained %	Major VOCs ^1^
Latent factors of VOCs (%):			
LEF-1	42.22	35.09	47
LEF-2	20.55	20.77	28
LEF-3	9.47	9.46	13
LEF-4	7.90	7.57	7
LEF-5	5.03	4.67	5
LEF-6	3.98	4.40	7
LEF-7	3.44	4.38	5
LEF-8	3.32	3.75	4
LEF-9	2.83	3.49	4
LEF-10	2.64	3.45	4
LEF-11	2.13	3.07	4
LEF-12	1.99	2.88	1
LEF-13	1.69	2.45	2
LEF-14	1.38	1.89	1
LEF-15	1.36	1.84	1
LEF-16	1.32	1.67	1
LEF-17	1.16	1.58	0

^1^ Number of VOCs with loadings >0.50 in absolute value.

**Table 4:** Loadings and communality (Com) of the 11 major latent explanatory factors of 129 volatile organic compounds (VOCs) expressed as relative percentage incidence on their total concentration in cooked meat patties (in bold: correlation coefficient over 0.5 or lower than −0.5).

VOC *m/z*:	Com	Loadings of the Latent Explanatory Factors:										
		LEF-1	LEF-2	LEF-3	LEF-4	LEF-5	LEF-6	LEF-7	LEF-8	LEF-9	LEF-10	LEF-11
26.016	0.96	−0.32	**0.70**	0.45	−0.12	−0.07	0.13	−0.13	0.11	0.04	−0.02	−0.10
28.032	0.78	−0.17	0.18	0.26	−0.34	−0.10	**0.51**	0.26	0.15	0.07	0.16	0.06
29.039	0.94	**0.59**	−0.06	0.12	−0.02	0.07	0.05	−0.04	−0.06	**0.71**	−0.16	−0.03
29.060	0.92	−0.02	**0.67**	0.41	−0.15	0.02	0.08	−0.03	0.02	0.44	−0.18	−0.18
31.019	0.90	−0.07	0.37	−0.01	0.00	−0.02	0.16	−0.08	−0.04	0.04	**0.74**	−0.10
33.034	0.72	**0.63**	−0.07	−0.15	0.23	0.04	−0.22	−0.01	0.02	−0.09	−0.04	−0.09
34.996	0.86	**0.56**	−0.32	−0.12	−0.23	0.00	0.05	0.31	−0.05	0.04	0.00	0.16
38.018	0.88	−0.16	0.38	**0.65**	−0.03	−0.05	0.15	−0.09	0.35	0.05	0.04	−0.04
41.038	0.96	−0.06	0.14	**0.94**	0.00	−0.07	0.01	−0.05	0.05	0.04	0.03	0.06
42.011	0.71	0.07	0.24	0.43	−0.07	−0.01	0.08	−0.12	0.04	0.05	−0.05	−0.45
42.034	0.76	0.45	−0.14	−0.36	−0.10	0.14	−0.24	0.24	0.06	−0.06	−0.10	−0.03
43.018	0.95	0.22	−0.12	−0.04	**0.83**	−0.01	−0.10	−0.18	−0.10	−0.06	0.12	−0.06
43.055	0.88	0.14	−0.11	**0.83**	0.15	−0.02	−0.06	−0.09	−0.09	0.15	−0.07	0.03
44.024	0.96	0.07	**0.77**	0.07	0.35	−0.03	0.01	−0.21	0.04	0.00	0.04	−0.21
46.996	0.82	−0.24	−0.35	−0.29	−0.35	−0.08	0.02	−0.05	−0.09	−0.11	−0.14	**0.52**
47.049	0.86	0.29	−0.10	−0.04	0.03	0.05	0.04	−0.05	−0.04	**0.84**	−0.14	−0.05
49.008	0.91	−0.42	−0.23	−0.31	−0.39	−0.08	0.04	−0.06	−0.08	−0.10	−0.10	**0.55**
52.028	0.88	0.19	0.28	−0.17	−0.24	0.14	0.00	−0.04	−0.06	0.01	−0.06	0.16
53.003	0.94	−0.13	0.48	**0.75**	−0.06	−0.05	0.01	−0.10	−0.01	0.06	0.08	−0.07
53.039	0.98	−0.42	**0.87**	−0.01	−0.16	−0.06	0.06	−0.06	0.01	−0.02	−0.01	−0.10
55.050	0.92	−0.32	0.37	−0.14	−0.20	−0.10	0.09	−0.08	0.06	−0.10	−0.09	**−0.72**
57.034	0.77	−0.12	0.45	**0.61**	−0.07	−0.10	0.10	−0.08	0.10	0.05	−0.02	−0.08
57.070	0.88	0.14	−0.24	**0.78**	−0.06	0.06	0.07	0.20	0.02	−0.09	−0.14	−0.03
59.967	0.83	0.19	−0.05	0.02	−0.30	−0.06	0.22	**0.73**	−0.02	0.03	−0.08	−0.11
60.053	0.93	0.43	−0.22	−0.07	0.08	−0.03	0.08	0.13	−0.07	−0.10	**0.80**	−0.02
61.035	0.91	**0.88**	−0.23	−0.02	0.10	0.06	−0.13	−0.02	−0.09	0.00	−0.06	0.04
62.023	0.83	0.35	−0.37	−0.26	0.14	0.02	−0.04	0.06	−0.07	0.13	0.13	0.05
63.026	0.83	0.20	−0.40	−0.31	0.08	−0.01	−0.05	0.01	−0.11	0.11	0.11	0.13
63.947	0.93	0.45	−0.29	0.05	−0.06	0.05	−0.03	**0.74**	0.15	−0.03	0.06	0.16
63.986	0.92	**0.82**	−0.20	−0.01	−0.05	0.06	0.09	0.28	0.00	0.07	0.03	−0.06
67.021	0.91	**−0.51**	0.07	−0.19	−0.29	−0.10	0.12	0.00	0.32	−0.09	−0.07	**0.58**
67.055	0.97	−0.34	**0.84**	0.07	−0.19	−0.04	0.09	−0.05	0.09	−0.02	−0.02	−0.12
67.992	0.81	−0.22	0.42	**0.65**	−0.13	−0.11	0.22	−0.03	0.24	−0.02	0.05	0.03
69.034	0.93	0.04	0.06	0.16	0.14	0.02	0.02	0.01	**0.92**	−0.02	−0.04	0.09
69.070	0.90	−0.44	**0.61**	0.15	−0.20	−0.05	0.08	−0.02	0.36	−0.03	−0.06	0.21
70.004	0.95	0.26	0.01	**0.90**	−0.05	−0.01	0.00	0.22	0.00	−0.06	−0.02	−0.01
71.015	0.76	0.11	0.08	**0.69**	0.42	−0.07	0.05	0.04	−0.01	−0.04	0.09	−0.08
71.049	0.76	−0.05	−0.19	0.10	**0.82**	−0.02	−0.05	−0.06	−0.11	−0.05	0.00	−0.02
71.085	0.90	0.07	−0.17	**0.85**	0.07	−0.04	−0.02	0.17	−0.04	−0.12	−0.08	−0.01
73.065	0.75	0.23	−0.39	−0.18	0.05	0.04	0.04	−0.03	0.19	0.45	0.22	0.12
75.028	0.93	**0.90**	−0.15	−0.02	0.10	0.12	−0.02	0.06	0.09	0.08	0.01	−0.03
75.044	0.85	**0.67**	−0.17	0.14	0.38	0.08	−0.09	−0.02	0.02	0.03	−0.04	−0.01
75.081	0.81	0.17	−0.22	**0.61**	−0.04	0.31	−0.06	0.04	0.08	0.00	−0.08	0.00
75.944	0.91	0.35	−0.25	0.13	−0.06	0.02	−0.08	**0.81**	−0.02	−0.05	0.03	0.07
77.016	0.95	**0.80**	−0.15	0.03	0.09	0.27	0.02	0.21	0.32	0.03	0.12	0.01
77.059	0.93	0.09	0.03	−0.11	0.04	−0.08	0.13	0.11	−0.05	−0.13	**0.90**	0.02
77.976	0.89	0.19	−0.13	0.06	−0.23	−0.05	0.21	**0.82**	−0.01	−0.01	0.16	−0.08
78.979	0.71	0.23	−0.01	−0.08	−0.04	**0.52**	−0.02	−0.06	0.21	0.01	−0.04	0.31
79.039	0.83	**0.60**	−0.13	−0.05	0.22	0.09	−0.04	0.03	0.02	0.02	−0.09	−0.08
79.055	0.97	0.29	−0.06	−0.01	−0.02	**0.93**	−0.02	0.01	−0.02	0.01	−0.06	−0.03
79.938	0.93	0.41	−0.26	0.11	−0.07	0.03	−0.07	**0.80**	−0.02	−0.05	0.03	0.07
80.041	0.94	**0.86**	−0.17	0.03	0.16	0.05	−0.04	0.15	0.02	0.01	−0.05	−0.06
81.038	0.93	**0.85**	−0.12	0.06	0.10	0.10	0.03	0.18	0.15	0.02	0.06	−0.02
81.071	0.96	−0.27	**0.91**	−0.01	−0.14	−0.03	0.09	−0.10	−0.03	−0.04	0.03	−0.07
82.047	0.85	**0.59**	0.17	0.07	−0.11	0.06	−0.01	0.19	0.22	0.13	0.10	0.00
84.044	0.95	0.42	0.04	0.05	0.04	0.03	0.15	0.03	**0.80**	0.06	−0.03	−0.25
85.014	0.81	0.24	−0.13	−0.01	−0.16	0.03	0.00	0.05	0.02	**0.73**	0.00	−0.03
85.073	0.75	0.29	**0.67**	−0.06	−0.07	0.01	0.15	−0.08	0.25	0.06	0.11	−0.26
85.101	0.91	0.05	−0.18	**0.84**	0.00	0.05	0.01	0.09	0.03	−0.08	−0.13	0.00
86.022	0.81	**0.71**	−0.16	−0.03	−0.08	0.06	−0.06	0.05	0.00	0.39	−0.04	−0.01
86.970	0.68	0.32	−0.11	0.10	0.29	−0.06	−0.19	0.15	**0.54**	−0.08	−0.08	−0.10
87.044	0.76	0.17	−0.10	0.00	**0.81**	0.01	−0.05	−0.10	0.18	−0.03	−0.01	−0.01
87.080	0.89	−0.07	0.29	0.01	0.19	0.02	−0.11	−0.12	**0.69**	0.00	−0.08	0.19
88.960	0.97	0.48	−0.25	0.01	**0.76**	0.04	−0.04	−0.08	0.26	0.01	0.00	−0.01
89.060	0.96	−0.07	−0.22	−0.04	**0.91**	−0.04	−0.05	−0.14	0.17	−0.04	0.00	0.02
91.059	0.90	0.47	−0.12	0.13	0.12	**0.75**	−0.04	−0.06	0.01	0.07	−0.03	0.02
93.069	0.71	0.37	−0.16	0.49	−0.06	0.19	−0.05	0.10	0.00	−0.02	0.08	0.05
95.019	0.55	**0.70**	−0.15	−0.04	−0.04	0.09	−0.06	0.01	−0.02	−0.02	−0.08	−0.01
95.053	0.87	**0.77**	−0.15	0.16	0.09	0.37	−0.05	0.07	0.05	0.03	−0.05	−0.02
95.088	0.74	0.12	**0.64**	−0.05	−0.17	0.07	0.29	−0.13	0.06	0.01	0.14	−0.33
97.064	0.69	−0.31	0.45	−0.07	0.03	0.02	0.30	−0.15	0.02	−0.08	0.20	0.02
97.101	0.80	−0.02	0.42	0.12	0.02	−0.05	**0.69**	0.11	0.01	0.07	0.14	−0.15
99.082	0.87	−0.36	**0.82**	0.03	−0.14	−0.06	0.08	−0.10	0.01	−0.02	0.06	−0.10
101.097	0.86	−0.48	**0.75**	−0.04	−0.17	−0.08	0.00	−0.04	0.03	−0.05	−0.02	−0.10
102.026	0.97	**0.95**	−0.12	−0.04	0.00	0.06	−0.03	0.08	−0.04	0.03	−0.03	−0.02
103.048	0.95	**0.93**	−0.21	−0.02	0.04	0.06	−0.05	0.07	−0.01	0.02	−0.02	0.03
105.041	0.93	**0.74**	−0.29	−0.04	0.46	0.15	−0.02	0.02	0.14	0.04	0.01	0.06
105.069	0.64	0.45	−0.07	−0.08	0.00	0.49	0.05	0.04	−0.03	0.20	0.18	−0.01
106.079	0.98	0.35	−0.05	−0.09	0.00	**0.91**	−0.05	0.00	−0.01	0.05	−0.02	−0.03
107.056	0.79	**0.50**	−0.11	−0.12	0.44	0.16	0.10	0.03	−0.04	−0.03	0.01	0.08
107.086	0.97	0.27	−0.06	−0.06	−0.02	**0.93**	−0.04	0.00	−0.01	0.01	−0.06	−0.03
109.076	0.87	**0.57**	−0.19	0.05	0.16	0.20	−0.19	−0.02	0.13	0.04	−0.06	0.00
109.103	0.90	**0.71**	0.22	0.01	−0.06	0.17	0.22	−0.04	−0.13	0.12	0.21	−0.07
110.969	0.81	**0.76**	−0.27	0.15	0.20	−0.02	0.03	0.16	0.02	0.05	0.03	−0.02
111.118	0.91	−0.07	**0.68**	0.02	−0.27	−0.03	**0.55**	0.02	0.01	0.05	0.10	−0.11
115.079	0.92	**0.91**	−0.05	0.00	0.09	0.08	0.10	0.12	0.14	0.08	0.01	−0.06
115.113	0.88	−0.08	**0.64**	0.01	−0.05	−0.02	**0.59**	0.04	0.11	0.08	0.22	−0.15
117.092	0.91	**0.81**	−0.11	−0.01	0.16	0.03	0.02	0.07	−0.06	0.05	0.33	−0.07
118.056	0.95	0.39	−0.28	−0.02	**0.84**	−0.01	−0.07	−0.06	0.05	−0.01	−0.02	−0.01
119.105	0.96	−0.41	**0.85**	−0.03	−0.15	−0.08	0.08	−0.04	0.03	−0.04	−0.03	−0.11
121.066	0.80	**0.83**	−0.19	−0.05	0.06	0.13	−0.05	0.04	−0.06	0.12	−0.06	0.01
121.105	0.88	**0.65**	0.36	−0.05	−0.03	0.30	0.00	0.01	0.01	0.08	0.05	−0.09
123.050	0.98	**0.95**	−0.19	0.02	0.09	0.08	−0.08	0.04	0.01	0.04	−0.03	0.00
123.114	0.92	**0.83**	0.00	0.09	0.06	0.08	0.08	−0.01	0.06	0.09	0.02	0.06
125.024	0.98	**0.96**	−0.14	0.00	0.03	0.07	−0.05	0.08	−0.06	0.00	−0.02	0.02
125.067	0.84	**0.64**	**0.57**	0.06	0.09	0.06	0.11	0.01	0.19	−0.01	−0.01	−0.02
125.097	0.90	−0.33	**0.87**	0.01	−0.01	−0.04	0.01	−0.11	−0.08	−0.09	0.03	0.04
125.132	0.90	0.10	**0.62**	0.13	−0.05	−0.04	**0.66**	0.00	−0.06	0.04	−0.05	0.15
127.081	0.94	**0.93**	−0.01	0.03	0.10	0.08	0.07	0.08	0.08	0.05	0.01	−0.05
127.113	0.89	**0.56**	0.49	−0.02	−0.08	0.02	0.14	−0.10	−0.16	0.01	0.15	−0.07
128.973	0.88	**0.89**	−0.16	0.12	−0.06	0.04	0.08	0.11	0.06	0.05	0.01	0.00
129.093	0.94	**0.77**	0.06	0.06	0.24	0.07	0.20	0.13	0.32	0.12	0.04	−0.11
129.128	0.93	0.04	0.45	0.01	−0.27	−0.05	**0.76**	0.12	0.00	0.14	0.17	−0.03
130.041	0.96	**0.92**	−0.23	0.01	0.17	0.07	−0.05	0.08	−0.02	0.04	−0.04	0.00
131.076	0.92	**0.68**	−0.21	0.02	**0.59**	0.07	0.02	0.00	0.14	0.02	0.00	−0.03
131.109	0.85	**0.87**	−0.04	−0.07	0.03	0.04	−0.02	0.01	−0.06	0.12	0.12	0.12
133.112	0.84	**0.56**	0.29	0.05	0.17	0.07	0.44	0.10	0.09	0.21	0.30	−0.03
134.975	0.73	0.48	0.06	−0.08	0.10	0.08	0.11	−0.11	0.06	0.05	−0.16	0.02
135.043	0.94	**0.94**	−0.15	0.01	0.06	0.06	−0.01	0.08	−0.01	0.00	0.15	−0.06
135.087	0.85	**0.90**	−0.16	0.01	0.03	0.06	0.00	0.10	0.02	0.02	0.04	0.00
137.067	0.98	**0.96**	−0.14	0.01	0.04	0.10	−0.01	0.08	0.04	0.06	−0.01	−0.02
137.132	0.56	**0.64**	−0.06	0.01	−0.14	0.07	−0.04	−0.14	−0.03	0.03	0.02	0.05
139.114	0.78	−0.30	**0.62**	−0.05	−0.02	0.03	0.22	−0.20	0.04	0.00	0.06	0.03
141.130	0.91	0.11	**0.78**	0.16	−0.16	−0.02	0.26	−0.08	−0.06	0.04	0.11	0.25
143.106	0.86	−0.30	**0.85**	0.03	0.02	−0.04	−0.02	−0.09	−0.09	−0.11	0.02	0.06
143.146	0.89	0.13	**0.62**	0.11	−0.02	−0.05	**0.66**	0.00	−0.07	0.03	−0.05	0.14
145.060	0.93	**0.88**	−0.09	0.06	0.31	0.17	0.01	0.10	0.06	0.02	−0.01	−0.01
147.130	0.82	0.05	0.36	−0.01	−0.07	0.08	0.33	−0.06	0.00	**0.68**	−0.03	0.05
151.120	0.94	**0.91**	−0.03	0.06	0.03	0.13	0.05	0.13	0.02	0.11	0.00	−0.06
153.131	0.75	**0.68**	0.33	0.03	−0.03	0.09	0.11	−0.10	−0.06	0.14	0.06	0.05
159.137	0.83	−0.32	**0.57**	−0.05	−0.06	−0.06	0.01	−0.05	−0.04	−0.10	**0.59**	0.09
160.899	0.87	**0.79**	−0.28	0.04	−0.15	0.00	−0.06	0.15	−0.07	0.02	−0.02	0.12
161.120	0.92	0.04	**0.90**	0.02	0.03	−0.02	0.21	−0.08	−0.05	−0.02	−0.04	0.03
165.161	0.80	0.19	**0.78**	0.00	−0.15	0.03	−0.05	0.11	0.00	0.07	0.01	0.13
173.148	0.81	**0.55**	0.33	−0.12	−0.11	0.03	0.12	−0.05	0.19	0.40	0.21	0.30
175.122	0.91	−0.08	**0.90**	−0.05	−0.08	−0.02	0.04	−0.05	0.06	0.00	−0.05	−0.21
187.169	0.93	−0.46	**0.72**	−0.04	−0.18	−0.05	−0.03	−0.02	0.12	0.01	0.04	0.37
201.182	0.97	−0.45	**0.83**	0.00	−0.12	−0.06	−0.05	−0.02	−0.02	−0.02	0.01	0.21
241.959	0.93	−0.20	**0.53**	**0.68**	−0.01	−0.04	0.12	−0.04	0.07	0.07	0.01	−0.14

## Supplementary Materials

The following are available online at https://www.mdpi.com/2304-8158/9/12/1738/s1, Table S1: Loadings and communality (Com) of minor latent explanatory factors (LEF) of 129 volatile organic compounds (VOCs) expressed as relative percentage incidence on their total concentration in cooked meat patties.
