# Supplementary material for: Rapid Profiling of the Volatilome of Cooked Meat by PTR-ToF-MS: Underlying Latent Explanatory Factors
Source: Foods. 2020 Nov 25;9(12):1738. doi: 10.3390/foods9121738 (PMC7768462; doi:10.3390/foods9121738)
Supplement: Supplementary file 1 [file foods-09-01738-s001.zip › Supplementary table S1_paper Foods LEF by Bittante et al.docx]

**Supplementary table S1** Loadings and communality (Com) of minor latent explanatory factors (LEF) of 129 volatile organic compounds (VOCs) expressed as relative percentage incidence on their total concentration in cooked meat patties (in bold the correlation coefficient over the 0.5 or lower the -0.5).

| *VOC m/z* | Loadings of the latent explanatory factors: | | | | | |
| --- | --- | --- | --- | --- | --- | --- |
|  | LEF-12 | LEF-13 | LEF-14 | LEF-15 | LEF-16 | LEF-17 |
| 26.016 | 0.19 | -0.05 | 0.19 | 0.01 | -0.04 | -0.07 |
| 28.032 | 0.09 | 0.25 | 0.25 | 0.04 | -0.03 | -0.03 |
| 29.039 | 0.14 | 0.01 | 0.02 | -0.02 | -0.04 | -0.10 |
| 29.060 | 0.03 | -0.04 | 0.04 | -0.05 | 0.01 | 0.00 |
| 31.019 | 0.32 | 0.04 | 0.10 | 0.09 | -0.21 | -0.02 |
| 33.034 | -0.31 | -0.01 | 0.15 | 0.10 | -0.12 | -0.19 |
| 34.996 | 0.10 | 0.45 | -0.07 | -0.15 | -0.01 | 0.04 |
| 38.018 | 0.31 | 0.06 | 0.06 | -0.01 | -0.05 | -0.12 |
| 41.038 | -0.03 | -0.09 | -0.05 | 0.02 | -0.03 | -0.15 |
| 42.011 | 0.28 | -0.27 | 0.27 | 0.01 | -0.03 | 0.01 |
| 42.034 | -0.17 | 0.25 | 0.17 | 0.27 | -0.18 | -0.02 |
| 43.018 | -0.05 | -0.01 | 0.14 | 0.03 | 0.30 | 0.01 |
| 43.055 | 0.06 | -0.06 | -0.16 | -0.01 | 0.02 | -0.25 |
| 46.034 | 0.12 | -0.21 | 0.26 | -0.04 | 0.09 | -0.04 |
| 46.996 | 0.33 | -0.01 | 0.03 | -0.05 | -0.07 | -0.01 |
| 47.049 | 0.12 | 0.03 | 0.00 | -0.02 | -0.04 | -0.12 |
| 49.008 | 0.25 | -0.13 | -0.03 | -0.01 | -0.09 | 0.06 |
| 52.028 | **0.76** | -0.10 | 0.14 | -0.02 | -0.12 | 0.02 |
| 53.003 | 0.24 | -0.13 | 0.06 | 0.05 | -0.08 | -0.12 |
| 53.039 | 0.03 | -0.06 | 0.05 | -0.03 | -0.01 | -0.03 |
| 55.050 | -0.03 | -0.21 | -0.08 | -0.08 | 0.00 | -0.03 |
| 57.034 | -0.15 | -0.26 | -0.05 | 0.00 | 0.02 | -0.21 |
| 57.070 | -0.20 | 0.02 | -0.03 | -0.01 | 0.04 | 0.26 |
| 59.967 | -0.06 | 0.03 | 0.26 | -0.09 | -0.13 | 0.00 |
| 60.053 | -0.04 | 0.07 | -0.04 | -0.05 | 0.02 | 0.05 |
| 61.035 | -0.16 | -0.09 | -0.01 | -0.05 | 0.02 | 0.08 |
| 62.023 | -0.05 | **0.65** | -0.03 | 0.01 | 0.07 | 0.00 |
| 63.026 | -0.16 | **0.66** | -0.08 | -0.01 | -0.03 | 0.01 |
| 63.947 | 0.09 | 0.08 | -0.14 | 0.01 | 0.07 | 0.03 |
| 63.986 | 0.23 | 0.21 | 0.03 | -0.02 | 0.04 | -0.01 |
| 67.021 | 0.21 | 0.06 | 0.00 | -0.06 | -0.04 | -0.04 |
| 67.055 | 0.07 | -0.18 | 0.13 | 0.00 | -0.06 | 0.02 |
| 67.992 | 0.06 | 0.06 | 0.00 | -0.05 | -0.06 | -0.11 |
| 69.034 | -0.04 | -0.01 | -0.11 | -0.03 | -0.02 | 0.07 |
| 69.070 | -0.01 | -0.22 | 0.22 | 0.05 | 0.01 | 0.02 |
| 70.004 | 0.02 | -0.06 | 0.04 | 0.03 | -0.07 | 0.08 |
| 71.015 | -0.09 | -0.01 | 0.14 | 0.02 | 0.17 | -0.03 |
| 71.049 | -0.07 | -0.02 | -0.03 | 0.03 | -0.07 | -0.04 |
| 71.085 | -0.22 | 0.03 | 0.01 | -0.06 | 0.01 | 0.19 |
| 73.065 | -0.16 | 0.20 | 0.25 | 0.16 | 0.12 | 0.19 |
| 75.028 | 0.24 | 0.04 | 0.05 | 0.05 | -0.02 | -0.01 |
| 75.044 | -0.15 | -0.08 | 0.15 | 0.15 | 0.33 | -0.02 |
| 75.081 | -0.04 | 0.07 | 0.00 | 0.09 | 0.06 | 0.48 |
| 75.944 | -0.03 | -0.02 | -0.17 | 0.07 | 0.06 | 0.02 |
| 77.016 | 0.16 | 0.07 | 0.01 | 0.09 | 0.04 | 0.02 |
| 77.059 | -0.16 | 0.05 | -0.06 | 0.01 | 0.07 | -0.01 |
| 77.976 | -0.04 | 0.03 | 0.14 | -0.06 | -0.01 | 0.00 |
| 78.979 | 0.43 | -0.19 | 0.05 | 0.04 | 0.09 | -0.04 |
| 79.039 | -0.13 | 0.04 | 0.07 | 0.00 | **0.59** | 0.06 |
| 79.055 | -0.01 | 0.01 | 0.01 | 0.05 | 0.01 | 0.00 |
| 79.938 | -0.03 | 0.03 | -0.16 | 0.06 | 0.05 | 0.03 |
| 80.041 | -0.13 | 0.05 | 0.03 | 0.04 | 0.31 | 0.00 |
| 81.038 | -0.08 | 0.16 | 0.00 | 0.15 | 0.21 | 0.03 |
| 81.071 | 0.05 | -0.05 | 0.03 | -0.04 | -0.05 | -0.03 |
| 82.047 | 0.15 | 0.25 | -0.08 | 0.48 | 0.12 | 0.02 |
| 84.044 | 0.13 | -0.04 | -0.05 | 0.02 | 0.03 | 0.02 |
| 85.014 | -0.04 | 0.16 | 0.06 | 0.05 | 0.00 | 0.36 |
| 85.073 | 0.18 | 0.00 | 0.08 | -0.01 | -0.01 | 0.02 |
| 85.101 | -0.15 | 0.02 | -0.04 | 0.01 | -0.03 | 0.32 |
| 86.022 | -0.08 | 0.18 | 0.01 | 0.07 | 0.00 | 0.22 |
| 86.970 | -0.17 | 0.08 | 0.22 | 0.00 | -0.02 | -0.11 |
| 87.044 | 0.02 | 0.07 | 0.06 | 0.11 | -0.09 | 0.02 |
| 87.080 | -0.05 | -0.22 | 0.23 | 0.34 | 0.04 | -0.04 |
| 88.960 | 0.09 | 0.01 | -0.03 | -0.10 | -0.04 | -0.01 |
| 89.060 | -0.08 | 0.01 | -0.07 | -0.07 | 0.02 | -0.01 |
| 91.059 | 0.11 | 0.00 | 0.01 | 0.05 | 0.05 | 0.20 |
| 93.069 | 0.10 | -0.11 | -0.13 | -0.07 | 0.01 | 0.45 |
| 95.019 | 0.01 | -0.01 | 0.11 | -0.05 | -0.05 | -0.01 |
| 95.053 | 0.07 | 0.00 | 0.04 | 0.20 | 0.02 | 0.17 |
| 95.088 | 0.03 | -0.16 | 0.00 | -0.05 | -0.08 | 0.08 |
| 97.064 | 0.01 | 0.02 | -0.12 | 0.43 | -0.14 | 0.03 |
| 97.101 | -0.02 | -0.01 | 0.22 | -0.10 | -0.07 | -0.08 |
| 99.082 | 0.04 | -0.07 | 0.01 | 0.01 | -0.04 | -0.08 |
| 101.097 | -0.02 | -0.05 | 0.01 | -0.01 | -0.02 | -0.08 |
| 102.026 | -0.07 | -0.04 | 0.05 | -0.02 | -0.12 | 0.04 |
| 103.048 | -0.08 | 0.05 | 0.02 | 0.00 | -0.13 | 0.01 |
| 105.041 | -0.03 | 0.08 | 0.12 | 0.10 | 0.02 | -0.02 |
| 105.069 | 0.04 | 0.06 | -0.01 | -0.28 | 0.00 | 0.17 |
| 106.079 | 0.04 | 0.01 | 0.02 | 0.01 | -0.02 | -0.02 |
| 107.056 | 0.09 | 0.03 | 0.46 | 0.02 | 0.24 | -0.02 |
| 107.086 | -0.04 | 0.02 | 0.01 | 0.04 | 0.00 | -0.04 |
| 109.076 | -0.07 | -0.08 | 0.12 | **0.59** | 0.09 | -0.01 |
| 109.103 | 0.15 | -0.20 | -0.12 | -0.18 | -0.24 | 0.10 |
| 110.969 | -0.02 | 0.24 | 0.03 | -0.07 | -0.03 | 0.00 |
| 111.118 | 0.03 | -0.09 | -0.13 | 0.05 | -0.07 | 0.09 |
| 115.079 | 0.06 | 0.03 | 0.01 | -0.02 | 0.06 | -0.06 |
| 115.113 | 0.08 | -0.06 | 0.09 | -0.02 | 0.07 | -0.09 |
| 117.092 | 0.02 | 0.03 | 0.02 | -0.03 | 0.32 | -0.02 |
| 118.056 | -0.08 | 0.03 | 0.00 | 0.01 | 0.02 | -0.01 |
| 119.105 | -0.08 | -0.07 | 0.00 | -0.05 | 0.05 | -0.07 |
| 121.066 | -0.07 | 0.03 | -0.02 | -0.02 | -0.15 | -0.07 |
| 121.105 | 0.44 | 0.03 | -0.06 | -0.01 | 0.10 | -0.04 |
| 123.050 | -0.05 | -0.04 | 0.02 | 0.07 | -0.02 | 0.03 |
| 123.114 | -0.04 | -0.16 | 0.00 | 0.40 | -0.08 | 0.06 |
| 125.024 | -0.06 | -0.02 | 0.01 | -0.04 | -0.14 | 0.05 |
| 125.067 | 0.10 | 0.07 | 0.02 | 0.06 | 0.12 | 0.00 |
| 125.097 | -0.02 | 0.03 | -0.04 | 0.03 | -0.06 | 0.03 |
| 125.132 | -0.02 | 0.01 | -0.06 | 0.08 | 0.04 | 0.04 |
| 127.081 | 0.11 | 0.06 | 0.03 | -0.01 | 0.10 | -0.02 |
| 127.113 | 0.00 | -0.31 | -0.11 | -0.20 | -0.29 | 0.13 |
| 128.973 | -0.05 | 0.03 | 0.04 | -0.05 | 0.00 | 0.06 |
| 129.093 | 0.21 | 0.14 | 0.00 | -0.02 | 0.13 | -0.09 |
| 129.128 | 0.03 | -0.09 | -0.06 | -0.03 | -0.07 | 0.04 |
| 130.041 | -0.05 | 0.02 | 0.09 | 0.06 | -0.01 | 0.03 |
| 131.076 | 0.06 | 0.05 | -0.06 | -0.04 | 0.13 | 0.03 |
| 131.109 | -0.17 | -0.02 | 0.06 | -0.03 | 0.04 | 0.06 |
| 133.112 | 0.03 | 0.01 | 0.03 | -0.09 | 0.16 | -0.11 |
| 134.975 | 0.23 | -0.16 | **0.59** | 0.03 | 0.03 | -0.01 |
| 135.043 | 0.06 | 0.01 | 0.02 | -0.02 | -0.01 | 0.03 |
| 135.087 | -0.04 | -0.01 | -0.04 | -0.01 | 0.04 | -0.02 |
| 137.067 | 0.04 | 0.00 | 0.00 | 0.07 | -0.02 | 0.03 |
| 137.132 | 0.11 | 0.00 | -0.07 | -0.20 | 0.18 | 0.05 |
| 139.114 | 0.10 | -0.09 | -0.28 | 0.31 | 0.04 | 0.14 |
| 141.130 | -0.02 | -0.12 | -0.15 | 0.03 | -0.19 | -0.01 |
| 143.106 | -0.05 | 0.06 | -0.02 | -0.01 | -0.06 | 0.02 |
| 143.146 | -0.02 | 0.00 | -0.06 | 0.05 | 0.05 | 0.00 |
| 145.060 | 0.01 | 0.03 | 0.03 | -0.02 | 0.05 | 0.02 |
| 147.130 | -0.13 | -0.09 | -0.15 | -0.03 | 0.09 | -0.22 |
| 151.120 | 0.20 | 0.06 | -0.03 | 0.07 | 0.09 | 0.03 |
| 153.131 | 0.19 | -0.04 | -0.17 | -0.02 | 0.01 | -0.21 |
| 159.137 | -0.04 | 0.01 | -0.10 | -0.03 | -0.05 | -0.05 |
| 160.899 | -0.08 | 0.19 | -0.07 | -0.15 | -0.15 | 0.10 |
| 161.120 | -0.12 | -0.03 | -0.09 | -0.02 | 0.10 | -0.10 |
| 165.161 | 0.22 | 0.15 | -0.09 | 0.14 | -0.03 | 0.05 |
| 173.148 | -0.02 | -0.06 | 0.07 | -0.05 | -0.05 | 0.12 |
| 175.122 | 0.02 | -0.09 | 0.01 | -0.11 | 0.06 | -0.06 |
| 187.169 | 0.05 | -0.03 | 0.07 | 0.04 | 0.03 | -0.05 |
| 201.182 | -0.01 | 0.09 | 0.00 | 0.03 | 0.01 | -0.05 |
| 241.959 | 0.22 | -0.03 | 0.04 | 0.07 | 0.04 | -0.18 |
